# Pioglitazone, a Peroxisome Proliferator-Activated Receptor-γ Agonist, Downregulates the Inflammatory Response in Cutaneous Leishmaniasis Patients Without Interfering in *Leishmania braziliensis* Killing by Monocytes

**DOI:** 10.3389/fcimb.2022.884237

**Published:** 2022-07-14

**Authors:** Maurício T. Nascimento, Ravena S. O. Cordeiro, Cayo Abreu, Camila P. Santos, Fábio Peixoto, Gabriela A. Duarte, Thiago Cardoso, Camila I. de Oliveira, Edgar M. Carvalho, Lucas P. Carvalho

**Affiliations:** ^1^ Laboratório de Pesquisas Clínicas, Instituto Gonçalo Moniz, FIOCRUZ, Salvador, Brazil; ^2^ Serviço de Imunologia, Complexo Hospitalar Prof. Edgard Santos, Universidade Federal da Bahia, Salvador, Brazil; ^3^ Programa de Pós-Graduação em Ciências da Saúde, Universidade Federal da Bahia, Salvador, Brazil; ^4^ Laboratório de Enfermidades Infecciosas Transmitidas por Vetores, Instituto Gonçalo Moniz, FIOCRUZ, Salvador, Brazil; ^5^ Ministério de Ciências e Tecnologia, Instituto Nacional de Ciências e Tecnologia-Doenças Tropicais, Salvador, Brazil

**Keywords:** cutaneous leishmaniasis (CL), PPAR-γ, pioglitazone, inflammation, monocytes

## Abstract

Patients with cutaneous leishmaniasis (CL) due to *Leishmania braziliensis* infection have an exacerbated inflammatory response associated with tissue damage and ulcer development. An increase in the rate of patients who fail therapy with pentavalent antimony has been documented. An adjuvant therapy with an anti-inflammatory drug with the potential of *Leishmania* killing would benefit CL patients. The aim of the present study was to investigate the contribution of peroxisome proliferator-activated receptor-γ (PPAR-γ) activation by pioglitazone in the regulation of the inflammatory response and *L. braziliensis* killing by monocytes. Pioglitazone is an oral drug used in the treatment of diabetes, and its main mechanism of action is through the activation of PPAR-γ, which is expressed in many cell types of the immune response. We found that activation of PPAR-γ by pioglitazone decreases the inflammatory response in CL patients without affecting *L. braziliensis* killing by monocytes. Our data suggest that pioglitazone may serve as an adjunctive treatment for CL caused by *L. braziliensis*.

## Introduction

Infection by *Leishmania braziliensis* causes a clinical spectrum of diseases in which cutaneous leishmaniasis (CL) is the most prevalent clinical form. CL lesions present an intense inflammatory infiltrate with the predominance of lymphocytes, mononuclear phagocytes, and a few parasites (Bittencourt and Barral, 1991; Saldanha et al., 2017). Although the inflammatory response is necessary to control parasite replication, an exaggerated production of pro-inflammatory cytokines induces tissue damage and the development of skin ulcers (Ribeiro-de-Jesus et al., 1998; Bacellar et al., 2002; Antonelli et al., 2005; Carvalho et al., 2007; Carvalho et al., 2012; Giudice et al., 2012; Novais et al., 2015; Santos et al., 2018; Amorim et al., 2019; Carvalho et al., 2020). In areas of *L. braziliensis* transmission, the use of pentavalent antimonial (Sb^V^) is the first drug of choice. However, an elevated rate of therapeutic failure rates has been reported, reaching 70% depending on the clinical form. Moreover, patients in the pre-ulcerative phase of the disease, as well as elderly subjects, are more likely to fail Sb^V^ therapy (Machado et al., 2002; Unger et al., 2009; Lago et al., 2018; Costa et al., 2018). Thus, the discovery of pharmacologic and immunological targets to decrease inflammation and parasite replication is highly important.

Previous studies have shown that the association between Sb^V^ and immunomodulatory drugs is beneficial for CL patients. The use of topical granulocyte macrophage-colony stimulating factor (GM-CSF) in association with standard doses (20 mg/kg/20 days) of Sb^V^ decreased healing time in refractory CL patients (Almeida et al., 1999). Pentoxifylline, a drug that decreases TNF production, associated with Sb^V^ was also more effective in the treatment of mucosal leishmaniasis patients when compared to Sb^V^ alone (Lessa et al., 2001; Machado et al., 2007).

Recently, studies have shown the possibility of using antidiabetic drugs to regulate the immune response in various types of inflammatory diseases such as CL (Carvalho et al., 2020), multiple sclerosis and metabolic syndrome (Negrotto and Mauricio, 2016), psoriasis (Singh and Anil, 2016), and diverticulitis (Freckelton et al., 2017). Pioglitazone and rosiglitazone are antidiabetic drugs from the family of thiazolidinediones (TZDs) that function as insulin sensitizers in peripheral and hepatic tissues by activating nuclear peroxisome proliferator-activated receptor-γ (PPAR-γ). PPAR-γ is a nuclear receptor expressed in several cell types of the immune response (e.g., monocytes, macrophages, dendritic cells, and lymphocytes), and signaling through PPAR-γ can regulate transcription factors such as nuclear factor kappa B (NF-κB) through ligand-dependent trans-repression mechanism (Aronoff et al., 2000; Ricote and Glass, 2007; Bouhlel et al., 2007; Wang et al., 2014; Zhu et al., 2016). The contribution of rosiglitazone and pioglitazone in the activation of PPAR-γ skews monocytes and macrophages to an M2 profile with enhanced anti-inflammatory properties (Bouhlel et al., 2007). Moreover, rosiglitazone activates PPAR-γ in murine bone marrow-derived macrophages, decreasing the production of pro-inflammatory cytokines in response to *Leishmania* lipophosphoglycan (LPG) (Lima et al., 2017). To date, the benefits of pioglitazone in the modulation of the inflammatory response and enhancement of parasite killing have not been evaluated in human CL.

Classically activated monocytes/macrophages produce pro-inflammatory soluble factors such as TNF, IL-1β, IL-6, and MMP9, while the alternative activation induces the expression of CD163, playing a pivotal role in wound healing (Porcheray et al., 2005; Giudice et al., 2012; Campos et al., 2014; Passos et al., 2015; Santos et al., 2018; Kasuya et al., 2018; Kim et al., 2019). CD163 is a haptoglobin–hemoglobin scavenger receptor present in monocytes/macrophages, and it is associated with the resolution of inflammation and the production of anti-inflammatory cytokines (Zwadlo et al., 1987; Högger et al., 1998; Kristiansen et al., 2001; Porcheray et al., 2005). The soluble form of CD163 (sCD163), however, is associated with inflammation, and sCD163 levels are increased in many inflammatory diseases (Kusi et al., 2008; Hassan et al., 2016; Endo et al., 2016; Silva et al., 2017; Nielsen et al., 2021). In this article, we show for the first time that exposure to pioglitazone skews monocytes from CL patients toward the alternative activation profile by decreasing the production of TNF, IL-1β, IL-6, MMP9, and sCD163, while it does not interfere in *Leishmania* killing. These findings suggest that pioglitazone is a strong candidate for adjuvant therapy in CL patients.

## Materials and Methods

### Subjects

This study was approved by the Institutional Review Board of the College of Medicine of Bahia (protocol no. 2.471.185) and the Brazilian Commission of Ethics in Research (2.512.434). All subjects provided written informed consent. This study was conducted in accordance with the Declaration of Helsinki and subsequent revisions. Fifteen CL patients were recruited from an endemic area of leishmaniasis—Corte de Pedra, Bahia-Brazil. Diagnostic criteria consisted of the presence of an ulcerated skin lesion, with no evidence of mucosal involvement, and the detection of *L. braziliensis* DNA by PCR (Cupolillo et al., 1994; Medzhitov and Janeway, 2000). A control group consisted of twelve healthy subjects (HSs) living in a non-endemic area of the same state without any reported exposure to *Leishmania*. All CL patients underwent clinical evaluations prior to the beginning of treatment.

### Parasite Cultures

An isolate of *L. braziliensis* (MHOM/BR/LTCP11245) was obtained from a skin lesion of a CL patient and identified as *L. braziliensis* by multilocus enzyme electrophoresis (Polari et al., 2019; Nascimento et al., 2021). Following isolation, parasites were cryopreserved in frozen nitrogen until use. Parasites were thawed and expanded in culture only once for this study. After selection, parasites were expanded in Schneider’s medium (Sigma-Aldrich, St Louis, MO, USA) supplemented with 20% heat-inactivated fetal bovine serum (FBS), 1% l-glutamine, penicillin (100 U/ml), and streptomycin (100 µg/ml) (Thermo Fisher Scientific, New York, NY, USA). *Leishmania* parasites in the stationary phase were used.

### Soluble *Leishmania* Antigen

Soluble *Leishmania* antigen (SLA) was prepared from an isolate of *L. braziliensis* as previously described (Reed 1986). Briefly, promastigotes were resuspended in lysis solution (Tris, HCL, EDTA, and leupeptin), immersed in liquid nitrogen, and subsequently thawed at 37°C. After the freeze–thaw cycle, parasites were sonicated and then centrifuged at 14,000 × *g*. The supernatant was filtered, and protein concentration and endotoxin levels (Limulus lysate test (Fisher Scientific, NY, USA)] were determined. The acceptable endotoxin concentration was 0 EU/ml. SLA was employed at 5 μg/ml in all assays.

### Peripheral Blood Mononuclear Cell and Biopsy Cultures

Peripheral blood mononuclear cells (PBMCs) were isolated from heparinized venous blood by Ficoll-Paque (GE Healthcare, Chicago, IL, USA) gradient centrifugation. After being washed in saline, the cells’ concentration was adjusted to 3 × 10^6^cells in 1 ml of RPMI-1640 (low glucose) (Thermo Fisher Scientific, NY, USA) supplemented with 10% FBS, penicillin (100 U/ml), and streptomycin (100 µg/ml) (Thermo Fisher Scientific, NY, USA). PBMCs were dispensed into 24-well plates and incubated at 37°C under 5% CO_2_ for 24 h in the presence or absence of SLA (5 µg/ml), lipopolysaccharide (LPS) (10 ng/ml), Pam3Cys (100 ng/ml), pioglitazone (1 μM) (Sigma-Aldrich), or GW9662 (10 μM) (Sigma-Aldrich).

CL patients and HSs were submitted to tissue biopsy using a 4-mm punch. Biopsied material was cultured in complete RPMI medium (low glucose) at 37°C and 5% CO_2_ for 72 h in the presence or absence of pioglitazone (1 μM) (Sigma-Aldrich). Cell culture and biopsy culture supernatants were collected and stored at −70°C until use. Cytokine levels were quantified by ELISA, and DNA levels (double stranded) were measured using a spectrophotometer. DNA extracted from the biopsy of patient CL was used as a positive control.

### Monocyte Cultures and *In Vitro* Infection

Monocytes were purified from PBMCs by negative selection using MACS columns (Miltenyi Biotec, Auburn, CA, USA). Monocytes were prepared following a method previously described by our laboratory to yield a purity of 99% and identified by flow cytometry as CD14+CD3−CD19− (Giudice et al., 2012). Briefly, monocytes (2.5 × 10^6^/ml) were maintained in RPMI-1640 (low glucose) (Thermo Fisher Scientific, NY, USA) supplemented with 10% FBS (Thermo Fisher Scientific, NY, USA), penicillin (100 U/ml), and streptomycin (100 µg/ml). After being washed, cells were infected with *L. braziliensis* (5:1) in the presence of pioglitazone (1 μM) at 37°C, 5% CO_2_. After 4 h, non-internalized parasites were removed by washing, and cells were incubated for 24 h. Parasite load was evaluated by optical microscopy, following H&E staining, by 3 different observers at 4 and 24 h post-infection.

### Cytokine Quantification

TNF, IL-6, IL-10 (BD Biosciences, San Jose, CA, USA), IL-1β, MMP9, and sCD163 (R&D Systems, Minneapolis, MN, USA) levels were determined by ELISA using commercial kits, following the manufacturers’ instructions. Results are expressed in pg/ml.

### *In Silico* Analysis

DrugBank v5 (https://go.drugbank.com/ ) was used to obtain the pharmacokinetics and pharmacodynamics information of pioglitazone (time to half-life 24 h). Protein–protein interaction (PPI) network analysis and Gene Ontology (GO) enrichment were performed using STRING v11.0 (https://string-db.org/). All interaction networks were limited to *Homo sapiens* species, and a minimum confidence score (high confidence: 0.700) was used for the interactions. The interaction networks that presented PPI enrichment p-value <0.05 were considered significant.

### Oxidative Burst Quantification

To evaluate the reactive oxygen species (ROS), 1 × 10^6^ monocytes (HSs) were treated with dihydrorhodamine-123 (DHR) at 10 ng/ml (Cayman Chemical Company, Ann Arbor, MI, USA) for 10 min. After that, cells were infected with *L. braziliensis* (5:1 ratio) for 24 h and then stained with anti-CD14 and anti-MHC-II fluorochrome-conjugated antibodies. The mean fluorescence intensity (MFI) of DHR in the cells was assessed by flow cytometry, and data were analyzed through FlowJo^®^.

### Cell Viability

PBMCs were isolated from heparinized venous blood by Ficoll-Paque (GE Healthcare, Chicago, IL, USA) gradient centrifugation. After being washed in saline, the cells’ concentration was adjusted to 3 × 10^6^ cells/ml of RPMI-1640 (low glucose) (Thermo Fisher Scientific, NY, USA) supplemented with 10% FBS, penicillin (100 U/ml), and streptomycin (100 µg/ml) (Thermo Fisher Scientific, NY, USA). PBMCs were dispensed into 24-well plates and incubated at 37°C under 5% CO_2_ for 24 h in the presence or absence of pioglitazone (1 μM) (Sigma-Aldrich). Monocytes were obtained as previously described. Briefly, 10 × 10^6^ PBMCs was dispensed into 6-well plates and maintained in RPMI-1640 (Thermo Fisher Scientific, NY, USA) supplemented with 10% FBS (Thermo Fisher Scientific, NY, USA), penicillin (100 U/ml), and streptomycin (100 µg/ml). Next, cells were washed two times with saline and maintained in RPMI-1640 (Thermo Fisher Scientific, NY, USA) supplemented with 10% FBS (Thermo Fisher Scientific, NY, USA), penicillin (100 U/ml), and streptomycin (100 µg/ml) for 24 h in the presence or absence of pioglitazone (1 μM).

Cell viability was determined by MTT. Briefly, 0.5 mg of Thiazolyl Blue Tetrazolium Bromide (Sigma-Aldrich) was added to 3 × 10^6^ cells, and cells were incubated for 4 h in the dark. The reaction was stopped, and the formazan salt crystals were solubilized by adding 10% dimethyl sulfoxide (DMSO) (Sigma-Aldrich). Next, the samples and blanks were transferred to 96-well flat-bottomed plates and read in a spectrophotometer at 570 nm. As a positive control, 3% paraformaldehyde (PFA) (Sigma-Aldrich) was used in all experiments.

### Statistical Analysis

All data obtained in *in vitro* assays were statistically analyzed using GraphPad Prism v8. All the results are shown as mean and SD. The paired t-test was used to compare two dependent continuous variables, whereas three or more dependent continuous variables were compared by the ANOVA test. The results were considered statistically significant when p < 0.05.

## Results

**Figure 1:**
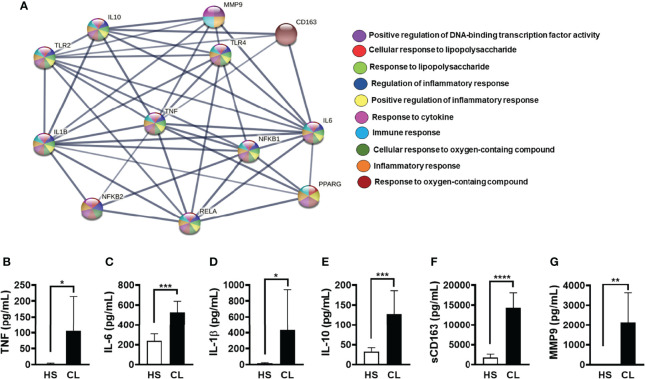
STRING protein–protein interaction (PPI) network. **(A)** Enrichment analysis of the Gene Ontology (GO) and pathways revealed that a group of inflammatory proteins that participate in the pathogenesis of cutaneous leishmaniasis (CL) (IL-1β, IL-6, TNF, NFκB1, and RELA) were directly linked to the peroxisome proliferator-activated receptor-γ (PPAR-γ) protein. The lines show the interaction between the proteins and their thickness and the level of interaction between them. The analysis shows the enrichment value PPI (p ≤ 1.0–16). Biopsies from CL patients (n = 10) and healthy subjects (HSs) (n = 5) were obtained with a 4-mm punch and cultured for 24 h. The levels of **(B)** TNF, **(C)** IL-6, **(D)** IL-1β, **(E)** sCD163 **(F)** MMP9, and **(G)** sCD163 were quantified by the ELISA technique. The box represents the mean, and the line above the box is the SD. Statistical analyses were performed using the paired t-test. *p < 0.05, **p < 0.01, ***p < 0.001, and ****p < 0.0001.

To investigate potentially relevant interactions of PPAR-γ, we performed a confidence analysis (high confidence 0.700) based on a PPI network, which allowed us to choose inputs (proteins) in addition to providing enrichment pathways (GO) of the generated interaction network. Briefly, we chose 12 proteins (TNF, IL-6, IL-1β, IL-10, MMP9, CD163, TLR2, TLR4, NFκB1, NFκB2, RELA, and PPARG) that are known to induce or downregulate the inflammatory response. We then identified five proteins (RELA, NFκB1, IL-1β, TNF, and IL-6) that directly interact with the nuclear receptor PPAR-γ. Furthermore, GO enrichment analysis showed that PPAR-γ is related to the following biological processes: upregulation of DNA-binding transcription factor activity, cellular response to LPS, inflammatory response, immune response, response to cytokine, and positive regulation of inflammatory response (**Figure 1A**). Although it is useful to identify general functional categories, protein network analysis and GO enrichment may be biased since an arbitrary selection of proteins can be used as the input. Therefore, we evaluated the ability of cells from biopsied CL lesions to produce TNF, IL-6, IL-1β, IL-10, sCD163, and MMP9. As expected, lesion cells produced higher levels of these mediators when compared to cells biopsied from HSs (**Figures 1B–G**). These findings suggest that PPAR-γ activation may be a key target to regulate the inflammatory response, characteristic of CL lesions.

Drug toxicity represents a major problem in the discovery of new therapeutic targets. In this way, we investigated whether pioglitazone could interfere with the viability of human cells through the MTT assay. We observed that pioglitazone did not alter the viability of PBMCs or monocytes (**Supplementary Figure 1**). To confirm that pioglitazone does not interfere with cell death, we measured DNA levels in the supernatants of cells obtained from HS biopsies and CL patients’ biopsies treated or not with pioglitazone. We observed that the CL lesion biopsies treated with pioglitazone did not differ in the amount of DNA when compared to the HS tissue. Furthermore, we observed that in CL lesion tissue, there is a >100-fold increase in the amount of DNA when compared to HS tissue (**Supplementary Figure 2**). It is important to emphasize that in CL lesions, there is a wide variety of dead cells (e.g., immune cells, bacteria, and parasites), which could possibly explain these results. Together, these results show that pioglitazone has no toxic effects in cells of CL patients.

**Figure 2:**
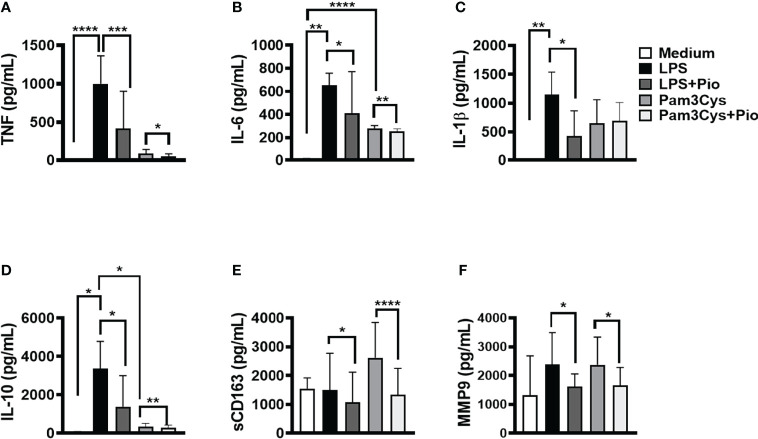
Pioglitazone downregulates immune response induced by TLR2 and TLR4 activation. Peripheral blood mononuclear cells (PBMCs) of healthy subjects (HSs) (n = 10) were cultured in the presence and absence of lipopolysaccharide (LPS) (10 ng/ml), Pam3Cys (100 ng/ml), or pioglitazone (1 µM) for 24 h. The levels of **(A)** TNF, **(B)** IL-6, **(C)** IL-1β, **(D)** IL-10, **(E)** sCD163 and **(F)** MMP9, were quantified by the ELISA technique. The box represents the mean, and the line above the box represents the SD. Statistical analyses were performed using the ANOVA test. *p < 0.05, **p < 0.01, ***p < 0.001, and ****p < 0.0001.

The signaling initiated by TLRs results in the NF-κB activation, cytokine production, and CD163 cleavage leading to the release of its soluble form (sCD163) (Medzhitov and Janeway, 2000). To test whether pioglitazone could interfere with NF-κB activation, we stimulated PBMCs from HSs with pioglitazone in the presence of LPS and Pam3Cys to induce TLR4 and TLR2 activation, respectively. Next, we assessed the levels of TNF, IL-6, IL-1β, IL-10, MMP9, and sCD163 produced. The presence of pioglitazone decreased the LPS-induced production of TNF, IL-6, IL-1β, IL-10, MMP9, and sCD163. Pam3Cys-activated monocytes also produced lower levels of TNF, IL-6, IL-10, MMP9, and sCD163 levels (**Figure 2**). These results suggest that PPAR-γ activation by pioglitazone inhibits NF-κB signaling, possibly regulating the inflammatory response.

**Figure 3:**
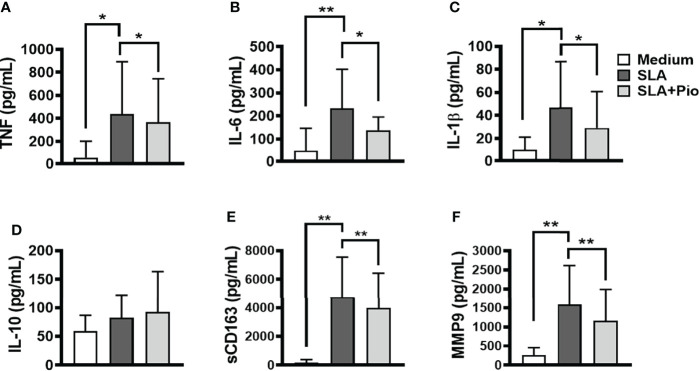
Pioglitazone downregulates the production of inflammatory mediators induced by *SLA*. Peripheral blood mononuclear cells (PBMCs) from cutaneous leishmaniasis (CL) (n = 15) were cultured in the presence or absence of soluble *Leishmania* antigen (SLA) (5 μg/ml) or pioglitazone (1 μM) for 24 h. The levels of **(A)** TNF, **(B)** IL-6, **(C)** IL-1β, **(D)** IL-10, **(E)** sCD163 and **(F)** MMP9, were quantified by the ELISA technique. The box represents the mean, and the line above the box is the SD. Statistical analyses were performed using the ANOVA test. *p < 0.05, **p < 0.01, and ***p < 0.001.

Recently, we showed that *L. braziliensis* induces the expression of TLR4 and TLR2 and the production of inflammatory cytokines by monocytes from CL patients (Polari et al., 2019). To test the hypothesis that pioglitazone can interfere in NF-κB signaling, thus decreasing the production of inflammatory cytokines, we stimulated PBMCs from CL patients with SLA, in the presence of pioglitazone. In the presence of SLA, pioglitazone-treated cells produced less TNF, IL-6, IL-1β, MMP9, and sCD163 but to our surprise did not alter IL-10 levels (**Figure 3**). These findings indicate that exposure to pioglitazone downmodulates the secretion of inflammatory mediators by monocytes from CL patients independently of IL-10.

**Figure 4:**
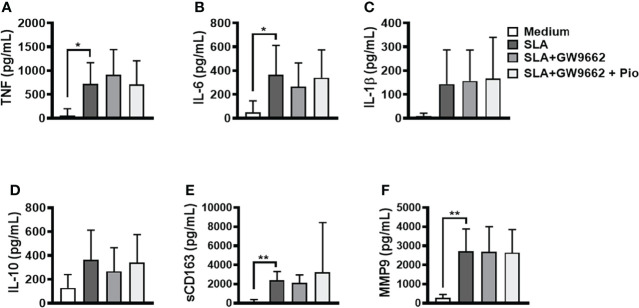
Blockage of peroxisome proliferator-activated receptor-γ (PPAR-γ) abrogates pioglitazone-mediated downregulation of inflammatory mediators induced by *SLA*. Peripheral blood mononuclear cells (PBMCs) from cutaneous leishmaniasis (CL) (n = 11) were cultured in the presence of GW9662 (10 μM) for 1 h and next stimulated with soluble *Leishmania* antigen (SLA) (5 μg/ml) or pioglitazone (1 μM) for 24 h. The levels of **(A)** TNF, **(B)** IL-6, **(C)** IL-1β, **(D)** IL-10, **(E)** sCD163 and **(F)** MMP9, were quantified by the ELISA technique. The box represents the mean, and the line above the box is the SD. Statistical analyses were performed using the ANOVA test.

Pioglitazone can reduce TLR2 and TLR4 expression in human monocytes, blocking NF-κB activation and cytokine production (Dasu et al., 2009; Kutsenko et al., 2012). To investigate whether pioglitazone affects SLA-stimulated PBMCs through PPAR-γ, we blocked PPAR-γ signaling with a selective and irreversible inhibitor and later stimulated these cells with SLA and pioglitazone (**Figure 4**). Our findings show that the downmodulation of inflammatory cytokine production by pioglitazone is completely dependent on PPAR-γ activity.

**Figure 5:**
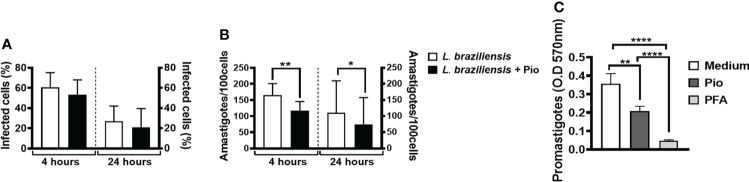
Pioglitazone has cytotoxic effects in *Leishmania braziliensis* parasites. Monocytes from healthy subjects (HSs) (n = 12) were cultured for 4 or 24 h in the presence or absence of *L. braziliensis* (5:1) and pioglitazone (1 μM). **(A)** The infection rate and **(B)** the number of amastigotes were determined by 3 different observers using optical microscopy. **(C)** *L. braziliensis* (n = 6) were cultured in the presence or absence of pioglitazone (1 μM) or paraformaldehyde (PFA) (3%) for 24 h. **(C)** OD of promastigotes form. The box represents the mean, and the line above the box is the SD. Statistical analyses were performed using the ANOVA test. *p < 0.05 and **p < 0.01.

Strong anti-inflammatory responses may contribute to the establishment of *Leishmania* infection (Malta-Santos et al., 2017). To investigate whether pioglitazone plays a role in *Leishmania* infection and survival, we first infected human monocytes in the presence or not of pioglitazone. Surprisingly, in the presence of pioglitazone, the percentage of infected cells was not altered after 4 or 24 h (**Figure 5A**). However, we observed a significantly reduced number of intracellular amastigotes at both time-points (**Figure 5B**). We also investigated whether pioglitazone could have a direct effect on the *Leishmania* killing. Pioglitazone considerably reduced the viability of *L. braziliensis* promastigotes (**Figure 5C**). These results suggest that PPAR-γ signaling is not directly involved in parasite entry, but, rather, pioglitazone has directly leishmanicidal activity and kills *Leishmania* parasites through PPAR-γ signaling.

**Figure 6:**
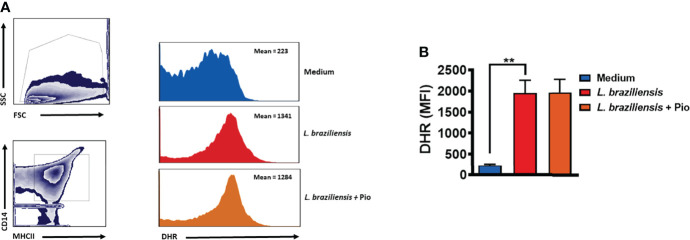
Pioglitazone does not interfere in *Leishmania braziliensis*-induced reactive oxygen species (ROS) production. Monocytes from healthy subjects (HSs) (n = 5) were treated with dihydrorhodamine-123 (DHR) at 10 ng/ml for 10 min and cultured for 24 h in the presence or absence of *L. braziliensis* (5:1) and pioglitazone (1 μM). Cells were stained with anti-CD14 and anti-MHC-II fluorochrome-conjugated antibodies. The mean fluorescence intensity (MFI) of the cells was assessed by flow cytometry. **(A)** Gate strategy representative plots and histograms. **(B)** Mean and SD from 5 HS. Statistical analyses were performed using the ANOVA t-test. *p < 0.05.

ROS production has been shown to be the main mechanism of *Leishmania* killing by human mononuclear phagocytes (Novais et al., 2014; Carneiro et al., 2016). To test whether pioglitazone interfered in ROS production, we infected monocytes with *Leishmania* in the presence and absence of pioglitazone and measured ROS levels. We did not observe a difference in ROS production comparing pioglitazone-treated and untreated *Leishmania*-infected monocytes (**Figure 6**). This result confirms that pioglitazone does not activate macrophages to kill *Leishmania*.

**Figure 7:**
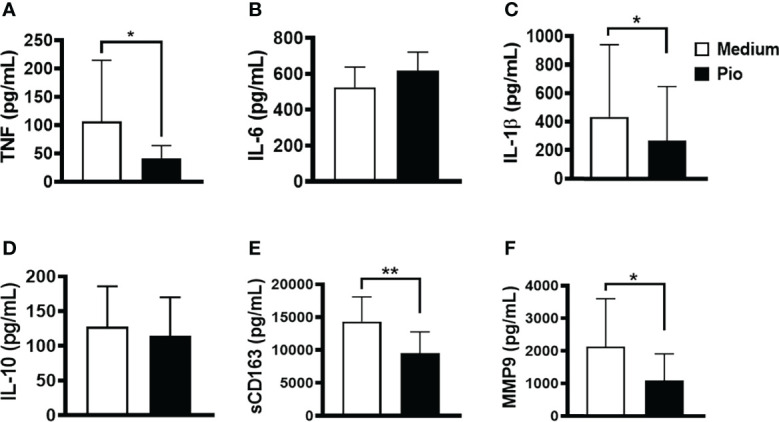
Pioglitazone down-regulates the production of inflammatory mediators in cells from CL lesions. Biopsies from CL patients (n=10) and HS (n= 5) were obtained with a 4mm punch and cultured for 24 hours. The levels of **(A)** TNF, **(B)** IL-6, **(C)** IL-1β, **(D)** IL-10, **(E)** sCD163 and **(F)** MMP9 were quantified by the ELISA technique. The box represents the mean and the line above the box the standard deviation. Statistical analyses were performed using the Paired t test *P < 0.05.

Monocytes are circulating cells in the peripheral blood that migrate CL lesions contributing to inflammation (Passos et al., 2015). Therefore, we evaluated whether exposure to pioglitazone can decrease the production of inflammatory cytokine production while maintaining the resolving phenotype characteristics of monocytes in cells from the lesion. Similar to the observations with PBMCs, we found that pioglitazone downregulated the production of TNF, IL-1β, and sCD163 but did not modify the production of IL-6 and IL-10 (**Figure 7**). These results show that pioglitazone exposure decreases inflammatory responses and may benefit CL patients.

## Discussion

In the last years, therapeutic failure rates in areas of *L. braziliensis* transmission have increased, reaching 70% depending on the clinical form and phase of the disease (Machado et al., 2002; Unger et al., 2009; Lago et al., 2018; Costa et al., 2018). Individuals infected with *L. braziliensis* develop an exaggerated inflammatory response that causes tissue damage leading to the appearance of skin ulcers (Ribeiro-de-Jesus et al., 1998; Bacellar et al., 2002; Antonelli et al., 2005; Campos et al., 2014; Novais et al., 2015; Passos et al., 2015; Santos et al., 2018; Amorim et al., 2019). Thus, the development of adjuvant therapies that can modulate the inflammatory response is desirable. Indeed, patients with mucosal leishmaniasis, the most inflammatory and severe form of tegumentary leishmaniasis, benefit from adjuvant therapy with pentoxifylline, a drug that decreases TNF production (Lessa et al., 2001). However, in CL patients, adjuvant therapy with pentoxifylline has not been shown to be effective, as it does not reduce healing time, suggesting that other molecules may be participating in the immunopathogenesis of CL (Brito et al., 2014). Pioglitazone is an oral drug used in the treatment of diabetes, and its main mechanism of action is to induce PPAR-γ. In this study, we investigated the effect of PPAR-γ activation by pioglitazone with regard to cytokine production by cells from CL patients. We found that exposure to pioglitazone downregulates the inflammatory response and does not interfere with the ability of monocytes to kill *L. braziliensis*.

Monocytes/macrophages secrete inflammatory mediators participating in the pathogenesis of CL. The activation profile and functions of these cells are affected by different cytokines, microbial products, and anti-inflammatory drugs. Thus, cytokines such as IFN-γ and TNF as well as LPS induce a “classic” activation profile (M1), whereas cytokines such as IL-4, IL-13, and IL-10 and the dexamethasone corticoid induce an “alternative” activation profile (M2) (Porcheray et al., 2005). In CL, a strong inflammatory response is observed, with high levels of IL-6, TNF, IL-1β, MMP9, and low levels of regulatory cytokines such as IL-10 (Bacellar et al., 2002; Campos et al., 2014; Passos et al., 2015; Santos et al., 2018; Carvalho et al., 2020), suggesting that mononuclear phagocytes are mostly classically activated (M1). Furthermore, it is important to emphasize that CL patients have a high frequency of intermediate monocytes (inflammatory monocytes, CD14+CD16+), a group of cells that show increased expression of TLR2/TLR4 and are the main source of inflammatory mediators such as TNF, IL-1β, and MMP9 (Campos et al., 2014; Passos et al., 2015; Santos et al., 2018; Polari et al., 2019). Here we show that the proinflammatory response (TNF, IL-6, IL-1β, MMP9, and sCD163), probably induced by the activation of TLR2 and TLR4 in human monocytes, is decreased when PPAR-γ is activated by pioglitazone. It has been documented that pioglitazone decreased the expression of MYD88, TLR2, TLR4, and NFκB1 (Dasu, 2009; Dana, 2019; Dana, 2020). These results support the notion that pioglitazone can interfere with NF-κB signaling favoring an environment where alternatively activated monocytes predominate, thus contributing to the modulation of the exacerbated inflammatory response observed in CL patients.

Alternately activated monocytes/macrophages decrease the inflammatory response by producing regulatory cytokines such as IL-10 (Sulahian et al., 2000; Porcheray et al., 2005). Furthermore, these cells are involved in debris clearance, tissue remodeling, and repair (Gordon, 2003; Xu et al., 2006; Gordon and Martinez, 2010; Sapudom et al., 2021). An important finding of our work was that activation of PPAR-γ by pioglitazone not only decreased the release of inflammatory mediators (TNF, IL-6, IL-1β, MMP9, and sCD163) but also maintained IL-10 production by PBMCs and by cells from lesion tissue of *L. braziliensis*-infected patients. The lack of effect of pioglitazone regarding IL-10 levels in SLA-stimulated PBMCs and lesion cells is somehow different from the results observed in LPS- and Pam3Cys-stimulated cells, where pioglitazone reduced IL-10 levels. The most likely explanation for this disparity is that *Leishmania* infection may induce cell types other than monocytes/macrophages to produce IL-10. Interestingly, although no decrease in IL-10 was detected, we found a lower parasite load in monocytes between 4 and 24 h of infection in the presence of pioglitazone. We thus evaluated whether pioglitazone treatment would interfere with ROS quantification. Pioglitazone also did not interfere in ROS production within infected monocytes. It has been shown that alternatively activated macrophages can produce ROS (Nandakumar et al., 2017), especially in the presence of exogenous PPAR-γ agonists (Lefèvre et al., 2010). Those observations caught our attention, as ROS production is the main mechanism that human monocytes/macrophages use to kill *L. braziliensis* (Novais et al., 2014; Carneiro et al., 2016). Also, the fact that a lower parasite load was observed 4 h after infection, in the presence of pioglitazone, indicates that this drug may interfere with direct *Leishmania* viability. Indeed, we found that pioglitazone significantly decreased the viability of *L. braziliensis in vitro*. Further studies will be carried out to investigate the mechanisms by which pioglitazone interferes with the viability of *Leishmania* during its interaction with monocytes.

Regarding the effects observed during the use of pioglitazone, it has been mainly reported that it increases insulin sensitivity, interference with lipid homeostasis, and regulation of the inflammatory response. Thus, many efforts have been made in the development of formulations for the topical use of pioglitazone (Silva-Abreu et al., 2017; Nair et al., 2019; Rojewska et al., 2020), opening new expectations for the treatment of skin diseases such as CL. Indeed, patients with psoriasis treated with topical pioglitazone had a decreased severity in disease score (Mittal et al., 2009; Singh and Anil, 2016). Therefore, the use of a topical formulation of pioglitazone would avoid systemic effects and would be a safer option in the case of CL. Altogether, our data show that PPAR-γ signaling induced by pioglitazone decreases the inflammatory response in CL patients without monocyte killing of *L. braziliensis*. In conclusion, our current work documents the advantage of activating PPAR-γ with pioglitazone, making it a good candidate for adjuvant CL immunotherapy.

## Data Availability Statement

The datasets presented in this study can be found in online repositories. The names of the repository/repositories and accession number(s) can be found in the article/**Supplementary Material**.

## Ethics Statement

The studies involving human participants were reviewed and approved by Institutional Review Board of the College of Medicine of Bahia (protocol no. 2.471.185) and the Brazilian Commission of Ethics in Research (2.512.434). The patients/participants provided their written informed consent to participate in this study.

## Author Contributions

All authors listed have made a substantial, direct, and intellectual contribution to the work and approved it for publication.

## Funding

This work was supported by the National Institutes of Health AI136862 and CAPES.

## Conflict of Interest

The authors declare that the research was conducted in the absence of any commercial or financial relationships that could be construed as a potential conflict of interest.

## Publisher’s Note

All claims expressed in this article are solely those of the authors and do not necessarily represent those of their affiliated organizations, or those of the publisher, the editors and the reviewers. Any product that may be evaluated in this article, or claim that may be made by its manufacturer, is not guaranteed or endorsed by the publisher.

## Supplementary Material

Supplementary Figure 1 Pioglitazone effect on PBMCs viability. PBMC and monocytes from HS (n=7) were cultured in the presence or absence of pioglitazone (1 uM) or PFA (3%) for 24 hours. Cells were treated within 0.5 mg/mL of Thiazolyl Blue Tetrazolium Bromide for 4 hours. The optical density (O.D) was assessed by read in a spectrophotometer at 570nm. **(A)** O.D of PBMC, **(B)** O.D of monocytes. The box represents the mean and the line above the box the standard deviation. Statistical analyses were performed using the ANOVA test ***P < 0.001 and ****P < 0.0001.

Supplementary Figure 2 Effects of pioglitazone on the death of tissue-derived cells. Biopsies from HS (n=5) and CL lesions (n=10) were obtained with a 4mm punch and cultured in the presence or absence of pioglitazone (1uM) for 24 hours. Purified DNA of CL lesion was used as positive control. The levels of dsDNA were quantified usinga spectrophotometer. The box represents the mean and the line above the box the standard deviation. Statistical analyses were performed using the ANOVA test *P < 0.05 and **P < 0.01.
