# Supplementary figures and images for: Pioglitazone, a Peroxisome Proliferator-Activated Receptor-γ Agonist, Downregulates the Inflammatory Response in Cutaneous Leishmaniasis Patients Without Interfering in Leishmania braziliensis Killing by Monocytes
Source: Front Cell Infect Microbiol. 2022 Jul 14;12:884237. doi: 10.3389/fcimb.2022.884237 (PMC9329526; doi:10.3389/fcimb.2022.884237)

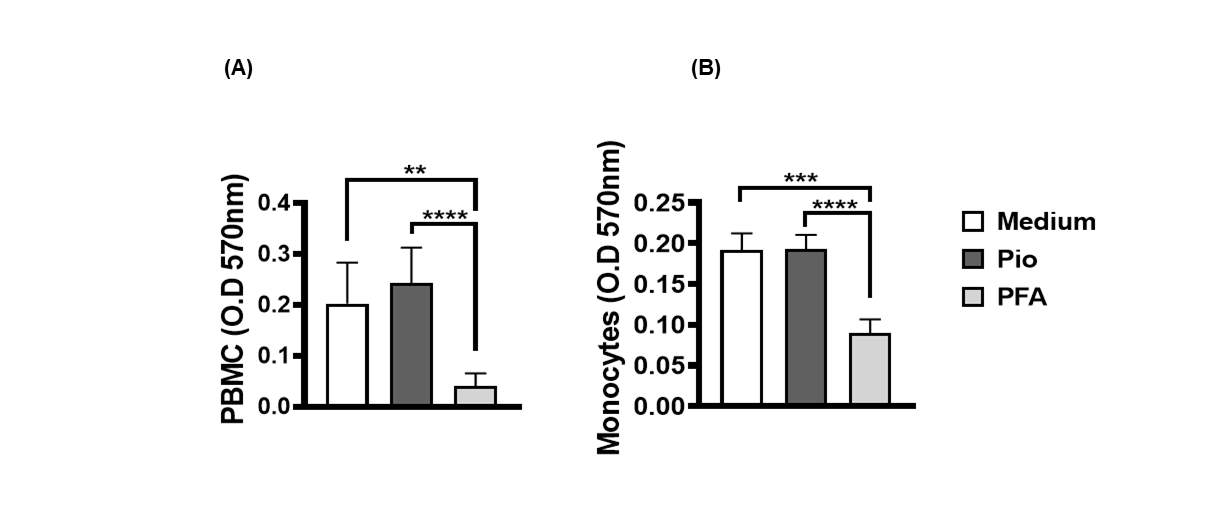

Supplement: Supplementary Figure 1 — Pioglitazone effect on PBMCs viability. PBMC and monocytes from HS (n=7) were cultured in the presence or absence of pioglitazone (1 uM) or PFA (3%) for 24 hours. Cells were treated within 0.5 mg/mL of Thiazolyl Blue Tetrazolium Bromide for 4 hours. The optical density (O.D) was assessed by read in a spectrophotometer at 570nm. (A) O.D of PBMC, (B) O.D of monocytes. The box represents the mean and the line above the box the standard deviation. Statistical analyses were performed using the ANOVA test ***P < 0.001 and ****P < 0.0001. [file Image_1.tif]

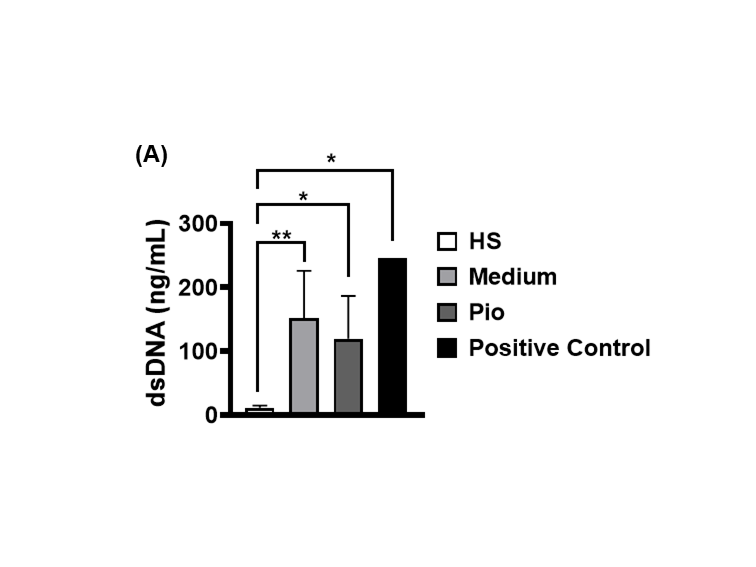

Supplement: Supplementary Figure 2 — Effects of pioglitazone on the death of tissue-derived cells. Biopsies from HS (n=5) and CL lesions (n=10) were obtained with a 4mm punch and cultured in the presence or absence of pioglitazone (1uM) for 24 hours. Purified DNA of CL lesion was used as positive control. The levels of dsDNA were quantified usinga spectrophotometer. The box represents the mean and the line above the box the standard deviation. Statistical analyses were performed using the ANOVA test *P < 0.05 and **P < 0.01. [file Image_2.tif]
